# Supplementary material for: Content-rich biological network constructed by mining PubMed abstracts
Source: BMC Bioinformatics. 2004 Oct 8;5:147. doi: 10.1186/1471-2105-5-147 (PMC528731; doi:10.1186/1471-2105-5-147)
Supplement: Additional File 2 — The original results of the above study (non-essential files are deleted to keep the file size under the limit set by BMC bioinformatics). [file 1471-2105-5-147-S2.bz2 › chilibotAdditionalFile2/dip20/602ID9751050E10981/html/CDK6_CDKN2A.html]

 


 **CDK6** and **CDKN2A** 
  
Found 170 abstracts in PubMed, retrieved 20.  
 

 What does Google say? 
 PDF only 
| .edu only 

---

**Interactive relationship** (e.g. stimulation, inhibition, etc)

**Inhibitory relationship**- Cyclin K resists the actions of the p16 INK4a  [ **CDKN2A** ]  and p27Kip1 inhibitors and extends the range of  **cdk6**  substrates, thereby inducing cell cycle progression toward S phase.  Ref: 12531804 Blood, 2003
- and 2 in vitro studies showed that the p16 INK4a  [ **CDKN2A** ]  allele from the BALB cJ mouse had a significantly decreased ability to bind and inhibit  **CDK6**  and to suppress cell growth when compared with the p16 INK4a  [ **CDKN2A** ]  allele from the A J mouse.  Ref: 12185599 Oncogene, 2002
- Aberrations in the MTS1  [ **CDKN2A** ]  tumor suppressor locus in oral squamous cell carcinoma lines preferentially affect the INK4A  [ **CDKN2A** ]  gene and result in increased  **cdk6**  activity.  Ref: 11854066 Oral Oncol, 2002
- The INK4  [ **CDKN2A** ]  family of cyclin dependent kinase CDK inhibitors negatively regulates cyclin D dependent CDK4 and  **CDK6**  and thereby retains the growth suppressive function of Rb family proteins.  Ref: 12556487 Mol Cell Biol, 2003
**Neutral relationship**- Most of the key interactions observed, were already anticipated in the analysis of the crystal structure of  **Cdk6**  p16 INK4a  [ **CDKN2A** ] .  Ref: 12437373 J Biomol Struct Dyn, 2002
- In order to get some insight into the key interactions governing recognition between different cyclin dependent kinases and the p16 INK4a  [ **CDKN2A** ]  tumor suppressor, the present work reports the results of molecular dynamics simulations of both, the  **Cdk6**  p16 INK4a  [ **CDKN2A** ]  complex and the Cdk4 p16 INK4a  [ **CDKN2A** ]  complex, respectively at 300 K.  Ref: 12437373 J Biomol Struct Dyn, 2002

**Non-interactive relationship** (e.g. studied together, co-existance, homology, etc.)

- In an attempt to model oral cancer in a human cell based system, we analyzed normal oral epithelial keratinocytes with the pRB pathway dysregulated by loss of expression of the cyclin dependent kinase cdk 4  **cdk6**  inhibitor p16 INK4A  [ **CDKN2A** ]  and or ectopic expression of cdk4 or expression of the human papillomavirus HPV type 16 E7 oncoprotein.  Ref: 12543805 Cancer Res, 2003
- Transition through this point requires  **cdk6**  4 cyclin D, since inhibition with TAT p16 INK4A  [ **CDKN2A** ]  during the first 3 to 5 h prevents cell cycle entry and maintains both naive and memory T cells in G 0.  Ref: 12640120 Mol Cell Biol, 2003
- Moreover, UV induced melanomas showed a strict reciprocal relationship between  **cdk6**  amplification and p16 INK4a  [ **CDKN2A** ]  loss, which is consistent with the actions of UV along the Rb pathway.  Ref: 12538879 Proc Natl Acad Sci U S A, 2003
- p16 Ink4a  [ **CDKN2A** ] , a CDK4 and  **CDK6**  specific inhibitor, failed to increase ROS levels, and cell cycle arrest induced by p16 was reversible following its down regulation, demonstrating the specificity of this p21 effect.  Ref: 11980715 EMBO J, 2002
- As shown by immunoblot analysis, EGCG treatment of LNCaP and DU145 cells resulted in significant dose and time dependent i upregulation of the protein expression of WAF1 p21, KIP1 p27, INK4a  [ **CDKN2A** ]  p16, and INK4c p18, ii down modulation of the protein expression of cyclin D1, cyclin E, cdk2, cdk4, and  **cdk6** , but not of cyclin D2, iii increase in the binding of cyclin D1 toward WAF1 p21 and KIP1 p27, and iv decrease in the binding of cyclin E toward cdk2.  Ref: 12559991 Arch Biochem Biophys, 2003
- In a melanoma model driven by H RAS activation and loss of p19 ARF  [ **CDKN2A** ]  function, UV exposure resulted in a marked acceleration in melanoma genesis, with nearly half of these tumors harboring amplification of cyclin dependent kinase cdk 6  [ **CDK6** ] .  Ref: 12538879 Proc Natl Acad Sci U S A, 2003
- Components upstream of the retinoblastoma tumour suppressor pRB, namely the INK4  [ **CDKN2A** ]  family of cyclin dependent kinase CDK inhibitors, the D type cyclins, their partner kinases CDK4  **CDK6** , and pRB as their critical substrate, are differentially targeted in diverse types of cancer.  Ref: 12754735 J Pathol< MedlineTA>J Pathol, 2003
- Structural Analysis of the Inhibition of Cdk4 and  **Cdk6**  by p16 INK4a  [ **CDKN2A** ]  through Molecular Dynamics Simulations.  Ref: 12437373 J Biomol Struct Dyn, 2002
- Surprisingly however, this could not explain arrest, since expression of mutant CDK4 and or  **CDK6** , incapable of binding p16 Ink4a  [ **CDKN2A** ] , did not confer any greater lifespan extension than the wild type CDKs.  Ref: 12082615 Oncogene, 2002
